# Impact of two sedation protocols on cardiac electrophysiology during spinal anesthesia

**DOI:** 10.55730/1300-0144.6182

**Published:** 2026-01-19

**Authors:** Selvinaz DURANTAŞ, Burak NALBANT, Fatma KAVAK AKELMA, Gökhan ERDEM, Abdulkadir BUT

**Affiliations:** 1 Clinic of Anesthesiology and Reanimation, Kızılcahamam State Hospital, Ankara, Turkiye; 2 Department of Anesthesiology and Reanimation, Faculty of Medicine, Ankara Yıldırım Beyazıt University, Ankara, Turkiye; 3 Department of Anesthesiology and Reanimation, Ankara Bilkent City Hospital, Ankara, Turkiye

**Keywords:** Spinal anesthesia, sedation, propofol, dexmedetomidine, iCEBc

## Abstract

**Background/aim:**

Perioperative factors such as anesthetic drugs, central blocks, surgical stress, pain, and anxiety can affect cardiac electrophysiology and increase the risk of arrhythmias. Arrhythmia susceptibility can be assessed using ventricular markers such as the QT interval (QT), heart rate-corrected QT interval (QTc), QT dispersion (QTd), time between peak and end of T wave (Tp–e), cardiac electrophysiologic balance index (iCEB), and heart rate-corrected index of cardiac electrophysiologic balance (iCEBc), obtained from noninvasive electrocardiography (ECG) data. This study compared the effects of propofol and dexmedetomidine sedation on these parameters in patients undergoing total knee arthroplasty performed under spinal anesthesia.

**Materials and methods:**

This prospective, observational clinical study was conducted at Ankara Bilkent City Hospital between March and August 2023, and included patients scheduled for elective total knee arthroplasty. Patients were divided into two groups based on the clinician’s preference: group P received propofol infusion, and group D received dexmedetomidine. All patients underwent spinal anesthesia. The primary outcome was the comparison of iCEB and iCEBc values between the two groups. Secondary outcomes included comparisons of QT, QTc, QTd, and Tp–e values.

**Results:**

A total of 74 participants were enrolled, and 70 were included in the final analysis (35 per group). No significant differences were found in demographic data, local anesthetic dose, spinal interventions, sensory block level, or bispectral index values (p > 0.05). QTc increased in both groups after spinal anesthesia; however, group P exhibited significantly higher values at t1 and t15 compared with group D (p = 0.004 and p = 0.046, respectively). iCEBc returned to baseline at t10 in group D and t45 in group P. A significant iCEBc increase from t0 to t1 was observed in group P but not in group D.

**Conclusion:**

Compared with propofol, dexmedetomidine sedation under spinal anesthesia was associated with differences in ECG-derived indices related to repolarization duration, homogeneity, repolarization–depolarization balance, and transmural distribution. These findings should be interpreted within the context of the study’s observational design and the use of ECG-derived markers rather than clinical arrhythmic endpoints.

## 1. Introduction

The administration of sedation during neuraxial anesthesia has been shown to enhance patient compliance with the anesthetic technique by reducing anxiety and to improve surgical conditions by increasing patient comfort [1–3]. Dexmedetomidine and propofol are among the most commonly used sedative agents during spinal anesthesia [3]. These agents are known to affect cardiac electrophysiology.

Spinal anesthesia induces a sympathetic blockade, leading to a shift in autonomic balance toward parasympathetic predominance. These changes, together with reflex responses to decreased venous return, can significantly influence heart rate and myocardial repolarization, thereby potentially altering electrocardiographic parameters. Spinal anesthesia can be safely administered when the requisite precautions are taken [4]. Furthermore, the choice of sedation is of particular importance, as agents such as propofol and dexmedetomidine have been reported to modulate cardiac ion channels, which may influence myocardial repolarization [5].

Electrocardiography (ECG) provides detailed information about cardiac electrophysiology when adequately interpreted [6]. In particular, the QT interval (QT), heart rate-corrected QT interval (QTc), QT dispersion (QTd), time between peak and end of T wave (Tp–e), cardiac electrophysiologic balance index (iCEB), and heart rate-corrected index of cardiac electrophysiologic balance (iCEBc) are significant ventricular arrhythmia markers in ECG analysis [6].

QT and QTc alone are insufficient to characterize nontorsadogenic ventricular arrhythmias [6,7]. The cardiac electrophysiological balance index (iCEB), previously described in the literature, has therefore been proposed as an additional ECG-derived marker. Calculated as the QT/QRS ratio, iCEB reflects the balance between ventricular repolarization and depolarization. Higher iCEB values have been associated with torsadogenic arrhythmias, whereas lower values are linked to nontorsadogenic ventricular arrhythmias, supporting its potential utility in differentiating drug-related electrophysiological effects [7,8].

We hypothesized that dexmedetomidine and propofol exert different effects on ECG-derived indices of cardiac electrophysiological balance, including iCEB and iCEBc, in patients undergoing surgery under spinal anesthesia. Accordingly, the primary objective of this study was to compare the effects of dexmedetomidine and propofol on iCEB and iCEBc values. Secondary objectives included comparisons of additional ventricular arrhythmia–related markers (QT, QTc, QTd, and Tp–e) as well as associated hemodynamic parameters between the two sedation regimens.

## 2. Materials and methods

The study was designed as a prospective, observational clinical study following approval from the local ethics committee (approval no: E2-22-2862; date: 23 November 2022). The study was registered at ClinicalTrials.gov (ID: NCT05757063). The study was conducted between March and August 2023 in the operating room of the Orthopedics Clinic at Bilkent City Hospital, Ankara, Türkiye. Written and verbal informed consent were obtained from all patients in accordance with the Declaration of Helsinki. Patients with American Society of Anesthesiologists (ASA) physical status I–II, aged 18–80 years, and with a body mass index (BMI) between 18 and 40 kg/m^2^ who were scheduled to undergo spinal anesthesia for total knee replacement surgery were included. Patients who exhibited an allergic response to the study drugs, those with a preoperative long QT interval of ≥460 ms, those taking medications known to affect the QT interval, patients with electrolyte abnormalities, hepatic or renal dysfunction, and patients with a history of heart disease were excluded. Furthermore, patients who required general anesthesia or additional medications (vasopressors, inotropes, or anticholinergics) during the surgical procedure were excluded. A total of 70 patients were included in this prospective observational study and were categorized into two groups according to the sedative regimen used in routine clinical practice: propofol (group P) or dexmedetomidine (group D).

Upon admission to the operating room, standard ASA monitoring (heart rate, noninvasive blood pressure, and oxygen saturation measured by pulse oximetry) and bispectral index (BIS) monitoring were applied. An isotonic saline infusion was administered at a rate of 5–10 mL/kg/h. The initial 12-lead electrocardiogram (ECG) was recorded using a GE Healthcare MAC 2000 system (GE Healthcare, Milwaukee, WI, USA) at a voltage calibration of 10 mV/mm and a paper speed of 25 mm/s (t0). The electrodes were positioned in proximity to the surgical field, and all recordings were obtained using the same placement. Following the initial electrocardiogram (ECG) recording, patients were placed in the seated position, and the L3–L4 intervertebral space was identified. A 25G spinal needle (Quincke spinal anesthesia needle; Egemen International Medical Devices Inc., İzmir, Türkiye) was introduced into the subarachnoid space. After confirmation of free cerebrospinal fluid flow, 12.5 mg of 0.5% hyperbaric bupivacaine was administered. Following spinal anesthesia, the second ECG (t1) was recorded during the loading-dose phase; therefore, t1 should be interpreted as a transitional time point with potential sedative influence. Supplemental oxygen was administered at 2 L/min via nasal cannula. The sedative agent was administered using an infusion pump, and the surgical field was subsequently covered with a sterile drape. At the 5th min of the spinal block, the level of sensory block was determined and recorded using the hot and cold test.

In the propofol group, a total dose of 1 mg/kg was administered over a 10 min period [9], followed by maintenance infusion at 2 mg/kg/h. In the dexmedetomidine group, a total dose of 0.5 μg/kg was administered over a 10 min period, followed by maintenance infusion at 0.5 μg/kg/h [10]. The target level of sedation was defined as a BIS value of 70–90, corresponding to light sedation with preserved responsiveness to verbal commands. ECG recordings were obtained at seven predefined time points: at baseline (t0) and at 1 (t1), 5 (t5), 10 (t10), 15 (t15), 45 (t45), and 75 (t75) min following spinal anesthesia [11]. Noninvasive blood pressure, pulse oximetry, heart rate, and BIS values were recorded at each time point. QT, QTc, iCEB, and iCEBc durations were calculated automatically using the calibrated ECG device and subsequently verified manually with a magnifying glass. For manual verification, the mean of three consecutive beats in leads D2 and V5 was used [6]. The iCEB value was derived by dividing the QT duration by the QRS duration (QT/QRS) [7]. The remaining parameters, including Tp–e and QTd, were calculated manually by a blinded investigator [6].

The QT duration was calculated as the interval between the onset of the Q wave and the end of the T wave, with values obtained by averaging three consecutive beats. Following measurement in each lead, the maximum difference between leads was recorded as QTd [12]. The Bazett formula (QT/√RR) was used to correct the QT duration for heart rate. The interval between the peak and end of the T wave was calculated from leads V5–V6 [12]. The end of the T wave was determined using the tangent method [6].

The primary outcome was the between-group difference in serial iCEB and iCEBc values measured at predefined time points (t0–t75) in patients undergoing surgery under spinal anesthesia.

The secondary outcomes were between-group differences in other ventricular arrhythmia–related ECG markers, including QT, QTc, QTd, and Tp–e, measured across the same time points, as well as associated hemodynamic variables (systolic blood pressure [SBP], diastolic blood pressure [DBP], mean arterial pressure [MAP], and heart rate [HR]).

### 2.1. Statistical analysis

A sample size calculation was performed using the statistical software G*Power version 3.1.9.7 (Franz Faul, Universität Kiel, Kiel, Germany), indicating that a minimum of 58 patients was required to achieve 80% power with an effect size of d = 0.66 and an alpha level of α = 0.05. A total of 74 patient datasets were collected, comprising 37 patients in each group.

Descriptive statistical methods, including frequency, percentage, mean, standard deviation, median, minimum, and maximum values, were used to summarize the data, and the chi-square (χ^2^) test was applied to evaluate differences in categorical variables. Normality was assessed using the Kolmogorov–Smirnov test, as well as evaluations of kurtosis and skewness, histograms, and box plots.

The independent-samples t-test was used for comparisons of normally distributed continuous variables, whereas the Mann–Whitney U test was applied for nonnormally distributed continuous variables.

Repolarization-related parameters (QTc, iCEBc, QT, QTd, Tp–e, and iCEB) measured repeatedly from t0 to t75 were analyzed using a mixed-design repeated-measures analysis of variance (ANOVA) with group and time as factors. Sphericity was assessed using Mauchly’s test and was not violated. When significant omnibus effects were detected, Bonferroni-adjusted post hoc pairwise comparisons were performed to identify differing time points (familywise α = 0.05 across 21 pairwise comparisons). Hemodynamic variables (SBP, DBP, MAP and HR) and secondary parameters (peripheral oxygen saturation [SpO_2_] and BIS) were compared between groups at each time point using the independent-samples t-test or the Mann–Whitney U test, as appropriate; multiplicity adjustment was not applied for these secondary time-point comparisons, and p values were interpreted conservatively. The level of statistical significance was set at α = 0.05. Data analyses were performed using IBM SPSS Statistics version 25.0 (IBM Corp., Armonk, NY, USA).

## 3. Results

**Figure 1:**
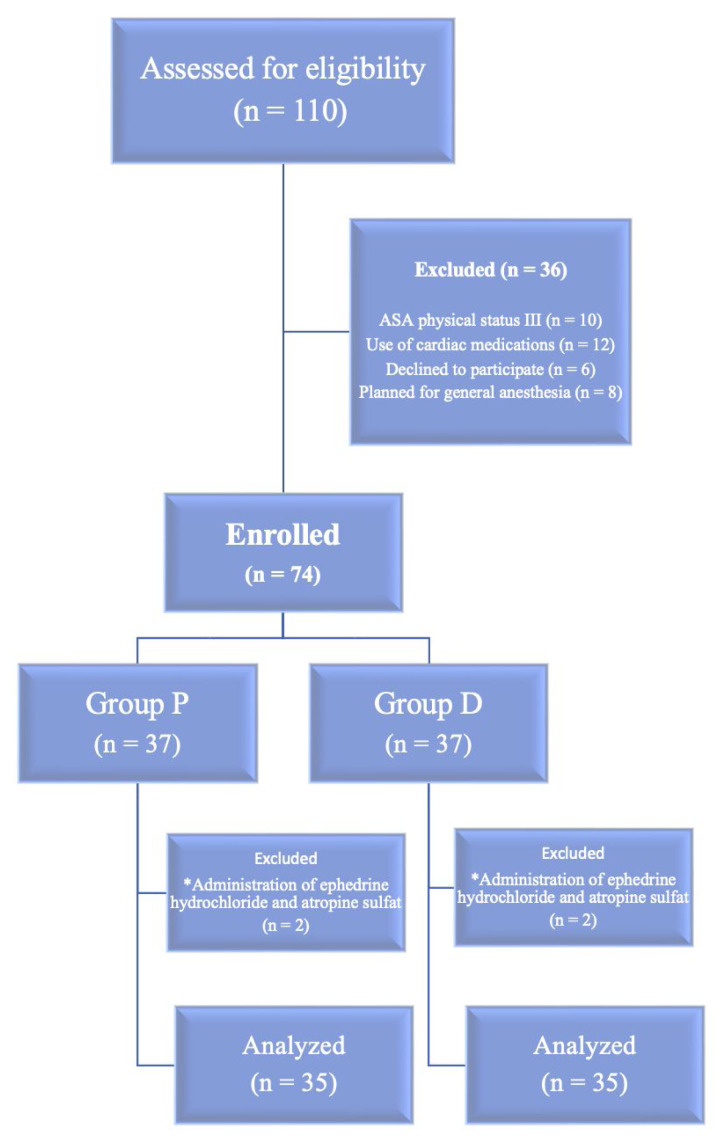
Participant flow diagram showing the numbers of participants screened, enrolled, excluded (with reasons), and included in the final analysis.

In total, 110 patients were assessed for eligibility. Of these patients, 36 were excluded (ASA physical status III, n = 10; use of cardiac medications, n = 12; refusal to participate, n = 6; planned general anesthesia, n = 8), and the remaining 74 patients were included and allocated to group P (n = 37) and group D (n = 37). Additionally, four patients were excluded from the analysis because of ephedrine and/or atropine administration (n = 2 per group), leaving 35 patients in each group for the final analysis (Figure 1). The two groups were comparable with respect to demographic characteristics, laboratory values, number of spinal interventions, amount of local anesthetic administered, and sensory block level (p > 0.05) (Table 1). Although group allocation reflected routine clinical practice rather than random assignment, this baseline balance may mitigate—though not eliminate—the risk of selection bias related to measured confounders. No significant differences were observed between the two groups with respect to oxygen saturation and BIS values (p > 0.05).

In group D, systolic and mean arterial blood pressures were significantly higher at time points t1, t5, t10, and t15; diastolic blood pressure was significantly higher at time points t5, t10, and t15; and mean arterial pressure was significantly higher at time points t1, t5, t10, and t15 (p < 0.05). Although the mean heart rate was lower in group D, this difference reached statistical significance only at t15 (p = 0.024) (Tables 2 and 3).

Significant differences were observed in repeated measurements of ventricular arrhythmia–related parameters, specifically QTc and iCEBc. As shown in Table 4, QTc values differed significantly between group P and group D at t1 (p = 0.004) and t15 (p = 0.046). No significant differences were observed among repeated QTc measurements in the intragroup analysis of group D (p = 0.211), whereas significant differences were observed in group P between t0–t1, t0–t5, t0–t10, t0–t15, t1–75, and t10–t75 (p < 0.001), indicating greater QTc stability in group D compared with group P (Table 4).

**Figure 2:**
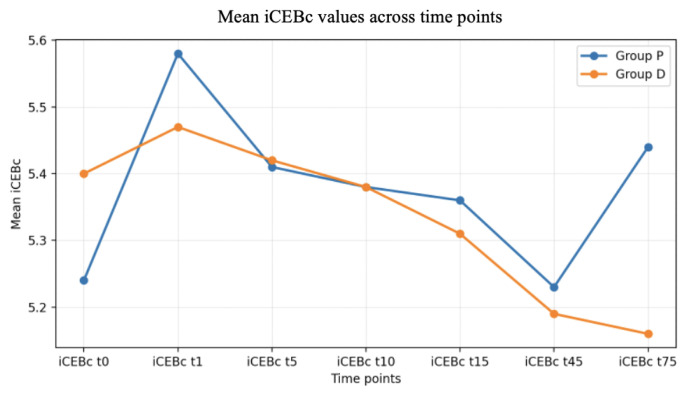
Changes in iCEBc over time in group D and group P during spinal anesthesia. iCEBc values are presented at predefined time points (t0–t75). Group D, dexmedetomidine; group P, propofol. Notably, iCEBc returned to baseline earlier in group D (t10) than in group P (t45). Abbreviations: iCEBc, heart rate-corrected index of cardiac electrophysiological balance.

No significant differences were observed in iCEBc scores between group P and group D across repeated measurements (p > 0.05). In the intragroup analysis, significant differences were observed in group P between t0 and t1 and between t1 and t45 (p = 0.001). In group D, significant differences were observed between t1 and t45, t1 and t75, and t5 and t75 (p = 0.001). Although a rapid increase in iCEBc was observed following spinal anesthesia in both groups, values subsequently approached baseline with the administration of sedation infusions. This return toward baseline occurred more rapidly in group D, with values approaching baseline at t10 and subsequently declining below baseline. In group P, values approached baseline at t45 and subsequently increased again (Figure 2).

Figure 2 illustrates distinct patterns of change in iCEBc measurements between the sedation groups. In the propofol group, a sudden and significant increase in iCEBc values was observed from t0 to t1, whereas no such increase was observed in the dexmedetomidine group.

QTd measurements differed significantly between group P and group D only at t45, with the mean QTd value being higher in group P than in group D at this time point (p < 0.036). Tp–e measurements differed significantly between group P and group D only at t75, with Tp–e values being higher in group P (p < 0.028). No significant intergroup differences were observed in QT values. The two groups exhibited a comparable pattern of change, with a notable increase in QT over time. Although increases between t10 and t15 and between t45 and t75 were not significant, increases at all other intervals reached statistical significance. At t15, iCEB values differed significantly between group P and group D (p < 0.049). During this interval, iCEB was higher in group D.

## 4. Discussion

We observed that both dexmedetomidine and propofol were associated with time-dependent changes in QTc and iCEBc during spinal anesthesia. Overall, QTc and iCEBc values appeared more stable over time in the dexmedetomidine group, whereas greater temporal variability in these ECG-derived indices was observed in the propofol group.

Given the potential advantages of spinal anesthesia, including reduced bleeding, preemptive analgesia, airway protection, and decreased airway manipulation, its use in cardiac patients is considered advantageous [13]. However, spinal anesthesia may also result in hemodynamic imbalances, sympathetic blockade, and reflex adrenergic responses above the level of blockade [11,13]. The block level remaining below the T4 dermatome causes compensatory sympathetic stimulation in the thoracic spinal nerves (T1–T4). It is hypothesized that this sympathetic stimulation represents the primary mechanism underlying QTc prolongation observed during spinal anesthesia [11]. In our study, the level of sensory block remained at T8 and below. It is generally assumed that sympathetic blockade occurs approximately two dermatomal levels above the sensory block. Accordingly, sympathetic blockade at T4 and above was unlikely to have occurred, and block levels were similar between the two groups. Consequently, the observed increases in QTc and iCEBc values in both groups are hypothesized to result from compensatory activation of thoracic sympathetic nerves following spinal anesthesia. Another potential reason may be the increase in catecholamine levels and sympathetic stimulation triggered by the spinal puncture. Nevertheless, we observed no significant difference in the number of spinal procedures performed in the two groups. Accordingly, no intergroup difference related to the number of spinal procedures was considered likely. The prolongation of QTc and the absence of a significant change in Tp–e with spinal anesthesia in our study are consistent with the findings of previous studies [11,14]. In a study involving preeclamptic pregnant women, a reduction in QTc values following spinal anesthesia was reported, in contrast to the present findings. This reduction was attributed to compensation for excessive sympathetic activity and increased systemic vascular resistance in patients with preeclampsia undergoing spinal anesthesia [15]. In another study involving pregnant women, no changes in QTc or Tp–e were observed following spinal anesthesia. However, the mean sensory block level reported in that study was T3 [16]. A reflex sympathetic response would not be expected to occur at these block levels following spinal anesthesia.

QTc prolongation was observed until the 15th min in the propofol group and until the 10th min in the dexmedetomidine group, after which QTc values decreased. Consistent with previous studies, the early QTc prolongation observed in the present study was attributed to spinal anesthesia [14]. QTc values returned to baseline earlier and more rapidly in group D than in group P and subsequently decreased below baseline in group D. In the intragroup analysis, no significant changes in QTc were observed in group D, indicating greater QTc stability in this group. Overall, spinal anesthesia was associated with increased reflex sympathetic activity and QTc prolongation, and compared with propofol, dexmedetomidine sedation was associated with a more rapid recovery of QTc values over time; however, given the observational study design and the absence of clinical arrhythmic outcome assessment, this association should not be interpreted as evidence of a preventive effect on major cardiac complications.

Similarly, iCEBc demonstrated a comparable pattern, exhibiting an increase in both groups following spinal anesthesia; however, this increase was more pronounced in group P. The significant increase in iCEBc between t0 and t1 in group P may indicate a transient alteration in repolarization–depolarization balance compared with baseline. Nevertheless, as clinical arrhythmic events were not evaluated as predefined study endpoints, the clinical implications of these changes should be interpreted cautiously. Overall, dexmedetomidine was associated with a more rapid return of iCEBc values toward baseline after spinal anesthesia, suggesting a shorter duration of iCEBc elevation compared with propofol. Given that the subsequent shortening was not statistically significant and remained within a limited range relative to baseline, these fluctuations are unlikely to be clinically meaningful; however, definitive conclusions regarding arrhythmic risk cannot be drawn from ECG-derived indices alone.

The findings of the present study were attributed to the vasoconstrictive effects of dexmedetomidine, which may compensate for the vasodilatory effects of spinal anesthesia and thereby attenuate reflex sympathetic responses originating from the thoracic spinal nerves. Accordingly, QTc prolongation resulting from reflex sympathetic responses following spinal anesthesia is likely to be limited. Although dexmedetomidine exhibits intrinsic dual effects, its primary hemodynamic response during initial loading doses has been shown to involve peripheral vasoconstriction and a hypertensive response in experimental studies using denervated heart models and control groups. In the study by Friesen et al., the primary hemodynamic response to dexmedetomidine was peripheral vasoconstriction accompanied by hypertension. This observation is consistent with the findings of the present study [17]. In the present study, blood pressure values were significantly higher in the dexmedetomidine group than in the propofol group during loading-dose administration.

The shortening effects of dexmedetomidine on QTc and iCEBc are also hypothesized to be related to its sedative and sympatholytic properties. Previous studies have demonstrated that dexmedetomidine administration is associated with reductions in plasma catecholamine levels [18]. Given that sympathetic activation and elevated catecholamine levels are known to prolong QTc and precipitate ventricular arrhythmias, the sympatholytic effects of dexmedetomidine and its associated reductions in catecholamine levels may contribute to antiarrhythmic effects, consistent with the present findings [19].

In contrast to our findings, the study conducted by Hammer et al. showed that dexmedetomidine administration in children undergoing ablation resulted in QTc prolongation [20]. This discrepancy may be attributed to the higher loading and infusion doses used in that study, as well as the inclusion of participants from different age groups. In another study, dexmedetomidine was administered using different dosing regimens, along with a control group, in patients undergoing general anesthesia. That study reached a similar conclusion, reporting that the dosing regimen used in the present study (0.5 μg/kg loading dose and 0.5 μg/kg/h maintenance dose) was associated with cardiac electrophysiological stability and shortening of QTc and iCEB [21]. In a study by Kim et al. involving patients receiving spinal anesthesia, an evaluation of QTc and Tp–e was conducted to compare the effects of dexmedetomidine sedation with a control group. The findings indicated that spinal anesthesia was associated with QTc prolongation until the 15th min, after which dexmedetomidine influenced a return toward baseline values. No significant effect on Tp–e was reported [11]. However, the potential influence of anxiety levels and associated sympathetic activation on cardiac electrophysiology was not addressed in the control group. In the present study, sedation was administered to both groups to mitigate anxiety and sympathoadrenal responses, with sedation initiated immediately following spinal anesthesia to evaluate the combined effects. The present findings suggest that the combination of spinal anesthesia and dexmedetomidine sedation may be associated with more favorable electrophysiological profiles related to ventricular arrhythmia risk.

A greater increase in QTc and iCEBc following spinal anesthesia was observed in the propofol group compared with the dexmedetomidine group. In addition, the return toward baseline was slower and less pronounced. These results suggest that the vasodilatory effect of propofol may not have adequately counteracted the reflex sympathetic response induced by spinal anesthesia. Despite these factors, propofol sedation may have partially attenuated QTc and iCEBc prolongation following spinal anesthesia through its sedative and sympatholytic effects, bringing values closer to baseline. Nevertheless, further studies with a control group are required to obtain definitive results. While the effects observed during the initial minutes likely reflect the combined influence of spinal anesthesia and sedation, the decrease in QTc at 15 min—possibly related to propofol—was consistent with findings from previous studies [22,23]. However, the tendency toward a return to baseline QTc values over time may also reflect attenuation of the hemodynamic effects of spinal anesthesia during the early postoperative period. This interpretation is supported by findings from an experimental study in guinea pigs, which demonstrated no significant effect of propofol on QTc [24]. The existing literature contains contradictory findings, with some studies reporting QTc prolongation associated with propofol, in contrast to the present results [25]. However, general anesthesia and different doses were used in these studies. It should be noted that the effects of propofol use in spinal anesthesia or solely for sedative purposes may differ. Although studies evaluating QT and QTc are available in the literature, to the best of our knowledge, no prior research has specifically examined iCEB and iCEBc in patients undergoing spinal anesthesia and/or sedation.

Although QTc and iCEBc differed statistically between groups at certain time points, the magnitude of these differences should be interpreted cautiously. Importantly, the observed changes did not appear to approach commonly used high-risk patterns for clinically meaningful proarrhythmic liability (e.g., marked absolute QTc prolongation or large within-patient QTc increases), and no clinical arrhythmic events were observed during the study period. Therefore, these findings are best interpreted as physiological differences in repolarization markers under two sedation regimens rather than definitive evidence of increased arrhythmic risk. We accordingly avoid causal or overgeneralized statements and emphasize the distinction between statistical significance and clinical significance.

This study has several limitations. First, because the sedative regimen was determined by routine clinical practice rather than random assignment, selection bias and residual/unmeasured confounding cannot be fully excluded. Nevertheless, the groups were broadly balanced with respect to key baseline and periprocedural variables (age, sex, BMI, electrolytes, hypertension, diabetes mellitus, number of spinal interventions, local anesthetic dose, and block level), which may mitigate—though not eliminate—confounding; accordingly, the findings should be interpreted as associative rather than strictly causal. Second, the study population comprised ASA I–II patients without known cardiac disease or baseline repolarization abnormalities, and dosing was kept near the lower end; therefore, the results may not be generalizable to higher-risk populations (e.g., congenital long QT syndrome, ischemic/structural heart disease, or marked autonomic dysfunction). In this context, dexmedetomidine-related bradycardia could be clinically consequential in patients with repolarization abnormalities. Notably, patients with long QT syndrome type 3 exhibit a bradycardia-triggered arrhythmia phenotype [26], and although dose reduction or titration to attenuate bradycardia may be advantageous, dexmedetomidine use may remain challenging in this population. Conversely, given its effects on QT, use in short QT syndrome could theoretically increase arrhythmic propensity. Third, plasma catecholamine levels were not measured, which may have influenced the evaluated parameters. Fourth, sensory block level was assessed only at the 5 min mark and no subsequent differences were observed, limiting inferences regarding later block dynamics. Finally, standardized effect sizes or precision estimates were not calculated for all time-point comparisons; therefore, the clinical relevance of small statistically significant differences should be interpreted cautiously.

In conclusion, dexmedetomidine sedation during spinal anesthesia in ASA I–II patients was associated with more favorable ECG-derived repolarization indices (reflecting repolarization duration and dispersion-related measures) compared with propofol. These electrophysiological differences may be clinically relevant in terms of perioperative cardiac stability, particularly in settings where sedative choice may influence myocardial repolarization dynamics during spinal anesthesia. Given the observational study design and the absence of clinical arrhythmic endpoints, the findings should be interpreted within their methodological context and should not be extrapolated to higher-risk cardiac populations.

## Figures and Tables

**Table 1:** Demographic and clinical characteristics of the study population.

Variables	Group P	Group D	p
**Age, year**	67.43 ± 6.07	67.43 ± 5.56	1.000
**Body mass index, kg/m** **^2^**	29.95 ± 3.88	30.37 ± 3.25	0.631
**Sex, Female/Male**	27 (77.1)/8 (22.9)	31 (88.6)/4 (11.4)	0.342
**Calcium, mEq/L**	9.59 ± 0.34	9.55 ± 0.99	0.831
**Magnesium, mEq/L**	1.94 ± 0.12	1.96 ± 0.16	0.638
**Potassium, mEq/L**	4.31 ± 0.29	4.23 ± 0.27	0.270
**Hypertension, n (%)**	21 (60.0)	25 (71.4)	0.450
**Diabetes mellitus n (%)**	11 (31.4)	11 (31.4)	1.000
**Number of spinal interventions**	1 [1–4]	1 [1–4]	0.483
**Local anesthetic dose, mg**	12.98 ± 1.07	13.38 ± 1.21	0.149
**Block level, T8/T10, n (%)**	18 (51.4)/17 (48.5)	16 (45.7)/19 (54.3)	0.811

Normally distributed data are presented as mean ± standard deviation.

Nonnormally distributed data are presented as median [minimum-maximum].

Categorical data are presented as number (%).

**Table 2:** Systolic and diastolic blood pressure values over time.

Time point	SBP group P (mean ± SD)	SBP group D (mean ± SD)	P value	DBP group P (mean ± SD)	DBP group D (mean ± SD)	p value
**t0**	159.40 ± 18.11	163.57 ± 20.64	0.372	84.11 ± 10.73	84.71 ± 10.77	0.816
**t1**	145.77 ± 17.73	154.20 ± 16.97	0.046*	78.20 ± 9.41	81.74 ± 11.05	0.153
**t5**	126.66 ± 19.00	143.37 ± 18.60	<0.001*	68.60 ± 9.79	76.46 ± 11.74	0.003*
**t10**	118.37 ± 20.34	134.03 ± 16.37	0.001*	64.03 ± 10.78	73.60 ± 11.14	0.001*
**t15**	115.46 ± 17.92	128.69 ± 18.76	0.004*	63.77 ± 10.13	69.17 ± 10.82	0.035*
**t45**	115.09 ± 17.27	119.11 ± 21.06	0.385	63.54 ± 10.12	65.17 ± 10.44	0.510
**t75**	119.34 ± 16.76	119.60 ± 23.42	0.958	63.83 ± 8.75	65.14 ± 11.72	0.597

Values are presented as mean ± standard deviation. SBP: systolic blood pressure; DBP: diastolic blood pressure.

* p < 0.05.

**Table 3:** Mean arterial pressure and heart rate values over time.

Time point	MAP group P (mean ± SD)	MAP group D (mean ± SD)	p value	HR group P (mean ± SD)	HR group D (mean ± SD)	p value
**t0**	112.97 ± 13.11	115.80 ± 12.01	0.350	79.77 ± 12.97	79.14 ± 11.98	0.834
**t1**	103.49 ± 10.89	110.74 ± 13.81	0.017*	82.29 ± 13.22	79.83 ± 14.77	0.466
**t5**	91.20 ± 11.83	103.17 ± 14.05	<0.001*	78.69 ± 13.49	73.43 ± 13.20	0.104
**t10**	85.03 ± 13.05	96.57 ± 12.13	<0.001*	72.03 ± 13.09	68.00 ± 10.09	0.154
**t15**	84.00 ± 11.47	92.97 ± 12.84	0.003*	71.00 ± 13.95	64.37 ± 9.62	0.024*
**t45**	84.26 ± 11.23	87.74 ± 13.32	0.241	61.69 ± 9.29	58.63 ± 7.42	0.133
**t75**	85.31 ± 11.73	86.69 ± 15.83	0.682	59.26 ± 8.02	57.54 ± 8.39	0.385

Values are presented as mean ± standard deviation. MAP: mean arterial pressure; HR: heart rate.

* p < 0.05.

**Table 4:** QTc measurements over time.

Measurements	Group P	Group D	p^a^
**QTc t0**	429.49 ± 22.48	436.54 ± 16.45	0.139
**QTc t1**	453.86 ± 23.38	439.03 ± 18.54	0.004^*^
**QTc t5**	447.37 ± 23.20	441.06 ± 20.26	0.229
**QTc t10**	447.80 ± 20.66	442.37 ± 24.18	0.316
**QTc t15**	448.66 ± 22.00	437.86 ± 22.38	0.046^*^
**QTc t45**	438.77 ± 23.02	432.09 ± 21.29	0.211
**QTc t75**	435.00 ± 23.37	430.91 ± 19.41	0.429
**p** **^b^**	0.000	0.211	
**Within group difference**	t0-t1, t0–t5, t0–t10, t0–t15, t1–t75 and t10–t75	*-*	

Values are presented as mean ± standard deviation. QTc: heart rate–corrected QT interval (ms); t: measurement time; p^a^: independent-samples t-test; p^b^*:* one-way repeated-measures ANOVA.

## References

[1] Sun S Wang J Bao N Chen Y Wang J Comparison of dexmedetomidine and fentanyl as local anesthetic adjuvants in spinal anesthesia: a systematic review and meta-analysis of randomized controlled trials Drug Design, Development and Therapy 2017 11 3413 3424 10.2147/DDDT.S146092 29238167 PMC5716323

[2] Kim DK Joo Y Sung TY Kim SY Shin HY Dreaming in sedation during spinal anesthesia: a comparison of propofol and midazolam infusion Anesthesia and Analgesia 2011 112 5 1076 1081 10.1213/ANE.0b013e3182042f93 21127282

[3] Kumakura Y Ishiyama T Matsuoka T Iijima T Matsukawa T Effects of spinal anesthesia and sedation with dexmedetomidine or propofol on cerebral regional oxygen saturation and systemic oxygenation a period after spinal injection Journal of Anesthesia 2020 34 6 806 813 10.1007/s00540-020-02816-5 32556601

[4] Pegu B Singh R Anesthetic management of a young primigravida a case of symptomatic long QT syndrome with a permanent pacemaker in-situ undergoing lower segment cesarean section delivery: A case report Saudi Journal of Anaesthesia 2023 17 2 256 259 10.4103/sja.sja_688_22 37260636 PMC10228844

[5] Stoetzer C Reuter S Doll T Foadi N Wegner F Inhibition of the cardiac Na(+) channel alpha-subunit Nav1.5 by propofol and dexmedetomidine Naunyn-Schmiedebergs Archives of Pharmacology 2016 389 3 315 325 10.1007/s00210-015-1195-1 26667357

[6] Postema PG Wilde AA The measurement of the QT interval Current Cardiology Reviews 2014 10 3 287 294 10.2174/1573403x10666140514103612 24827793 PMC4040880

[7] Robyns T Lu HR Gallacher DJ Garweg C Ector J Evaluation of Index of Cardio-Electrophysiological Balance (iCEB) as a New Biomarker for the Identification of Patients at Increased Arrhythmic Risk Annals of Noninvasive Electrocardiology 2016 21 3 294 304 10.1111/anec.12309 26305685 PMC6931457

[8] Askin L Tanriverdi O Evaluation of index of cardio-electrophysiological balance in patients with coronary slow flow Acta Cardiologica 2022 77 4 337 341 10.1080/00015385.2021.1945232 34218730

[9] Ata F Yavaşçaoğlu B Girgin N Yılmaz C Kaya F Comparison of the effects of propofol and dexmedetomidine sedation on axillary block Kafkas Journal of Medical Sciences 2016 6 1 1 7 10.5505/kjms.2016.27928

[10] Tasbihgou SR Barends CRM Absalom AR The role of dexmedetomidine in neurosurgery Best Practice and Research: Clinical Anaesthesiology 2021 35 2 221 229 10.1016/j.bpa.2020.10.002 34030806

[11] Kim Y Kim SY Lee JS Kong HJ Han DW Effect of dexmedetomidine on the corrected QT and Tp-e intervals during spinal anesthesia Yonsei Medical Journal 2014 55 2 517 522 10.3349/ymj.2014.55.2.517 24532526 PMC3936610

[12] Whyte SD Booker PD Buckley DG The effects of propofol and sevoflurane on the QT interval and transmural dispersion of repolarization in children Anesthesia and Analgesia 2005 100 1 71 77 10.1213/01.ANE.0000140781.18391.41 15616054

[13] Butterworth J Physiology of spinal anesthesia: what are the implications for management? Regional Anesthesia and Pain Medicine 1998 23 4 370 373 9690588 10.1016/s1098-7339(98)90008-6

[14] Owczuk R Sawicka W Wujtewicz MA Kawecka A Lasek J Influence of spinal anesthesia on corrected QT interval Regional Anesthesia and Pain Medicine 2005 30 6 548 552 10.1016/j.rapm.2005.06.005 16326340

[15] Sen S Ozmert G Turan H Caliskan E Onbasili A The effects of spinal anesthesia on QT interval in preeclamptic patients Anesthesia and Analgesia 2006 103 5 1250 1255 10.1213/01.ane.0000247965.41868.da 17056963

[16] Guillon A Leyre S Remerand F Taihlan B Perrotin F Modification of Tp-e and QTc intervals during caesarean section under spinal anaesthesia Anaesthesia 2010 65 4 337 342 10.1111/j.1365-2044.2010.06246.x 20136804

[17] Friesen RH Nichols CS Twite MD Cardwell KA Pan Z The hemodynamic response to dexmedetomidine loading dose in children with and without pulmonary hypertension Anesthesia and Analgesia 2013 117 4 953 959 10.1213/ANE.0b013e3182a15aa6 23960035 PMC3830564

[18] Kang R Jeong JS Ko JS Lee SY Lee JH Intraoperative dexmedetomidine attenuates norepinephrine levels in patients undergoing transsphenoidal surgery: a randomized, placebo-controlled trial BMC Anesthesiology 2020 20 1 100 10.1186/s12871-020-01025-7 32359367 PMC7195722

[19] Manolis AA Manolis TA Apostolopoulos EJ Apostolaki NE Melita H The role of the autonomic nervous system in cardiac arrhythmias: The neuro-cardiac axis, more foe than friend? Trends in Cardiovascular Medicine 2021 31 5 290 302 10.1016/j.tcm.2020.04.011 32434043

[20] Hammer GB Drover DR Cao H Jackson E Williams GD The effects of dexmedetomidine on cardiac electrophysiology in children Anesthesia and Analgesia 2008 106 1 79 83 10.1213/01.ane.0000297421.92857.4e 18165557

[21] Tan C Yan S Shen J Wu H Yu L Effects of dexmedetomidine on cardiac electrophysiology in patients undergoing general anesthesia during perioperative period: a randomized controlled trial BMC Anesthesiology 2022 22 1 271 10.1186/s12871-022-01811-5 36008759 PMC9404616

[22] Tominaga S Terao Y Urabe S Ono M Oji N The effects of intravenous anesthetics on QT interval during anesthetic induction with desflurane JA Clinical Reports 2018 4 1 57 10.1186/s40981-018-0195-9 32025881 PMC6967065

[23] Oji M Terao Y Toyoda T Kuriyama T Miura K Differential effects of propofol and sevoflurane on QT interval during anesthetic induction Journal of Clinical Monitoring and Computing 2013 27 3 243 248 10.1007/s10877-012-9420-7 23242843

[24] Yamada M Hatakeyama N Malykhina AP Yamazaki M Momose Y The effects of sevoflurane and propofol on QT interval and heterologously expressed human ether-a-go-go related gene currents in Xenopus oocytes Anesthesia and Analgesia 2006 102 1 98 103 10.1213/01.ANE.0000184257.54917.99 16368812

[25] Kim DH Kweon TD Nam SB Han DW Cho WY Effects of target concentration infusion of propofol and tracheal intubation on QTc interval Anaesthesia 2008 63 10 1061 1064 10.1111/j.1365-2044.2008.05564.x 18717665

[26] Booker PD Whyte SD Ladusans EJ Long QT syndrome and anaesthesia British Journal of Anaesthesia 2003 90 3 349 366 10.1093/bja/aeg061 12594150

